# Supplementary material for: Stress-induced plasticity of dynamic collagen networks
Source: Nat Commun. 2017 Oct 10;8:842. doi: 10.1038/s41467-017-01011-7 (PMC5635002; doi:10.1038/s41467-017-01011-7)
Supplement: Supplementary file 1 — Supplementary Information [file 41467_2017_1011_MOESM1_ESM.pdf]

Supplementary Methods

Additional details of characterizations of collagen bundles

Relative intensity increase $\Delta F/F$ We have calculated the relative intensity increase $\Delta F/F$ to quantify the densified collagen fibers at the collagen bundle. Supplementary Fig. 1 demonstrates the image analysis procedures to calculate $\Delta F/F$. A typical confocal image (1024 X 1024 pixels or 366 X 366 μm) is shown in Supplementary Fig. 1A. The region highlighted by the green box in (A) is magnified in (B). As demonstrated in (C), a rectangular region (enclosed in red) is manually selected to calculate the mean intensity of the collagen bundle F_b. We then create a mask to enclose the region near the cells and the collagen bundle. To compute the background intensity fluctuations, 500 circular patches are sampled at random positions outside the mask. Each circle has the same area as the bundle region in (B), and the mean intensity F_i of each circular patch is calculated. The relative intensity used in the main text is defined as $\Delta F/F = \frac{F_b - <F_i>}{<F_i>}$.

Bundle formation and irreversibility $\Delta F/F$ increases after cell seeding, indicating the cellular traction force in action to form collagen bundles. Collagen bundles are plastic deformations, and may persist even with the release of cellular traction force. Supplementary Fig. 4 shows examples of temporal profiles of $\Delta F/F$ and irreversible deformations.

cell-cell distances and cell morphology The relative intensity increase $\Delta F/F$ negatively correlates with the cell-cell distance, as shown in Supplementary Fig. 2A. We find that formation of

collagen bundle is robust. To better demonstrate this, we distinguish each collagen bundle based on the signal to noise ratio (SNR) of its reflectance intensity. In Supplementary Fig. 2A, closed symbols represent $\Delta F/F > 3\delta F/F$ (corresponding to $p < 0.0027$), and open symbols represent $\Delta F/F < 3\delta F/F$. Here δF is the background fluctuation that follows closely to Gaussian distribution. It is evident that after enough maturation (1 hour), collagen bundles with high SNR form between cells as far as 80 μm apart. This is to be compared with the average cell size (long-axis) of 17.2 $\pm$2.6 μm and the matrix pore size (radius of the maximumly fitting circle) of 3.0$\pm$0.7 μm.

At the same time, most cells we have analyzed that form collagen bundles have rounded morphology (Supplementary Fig. 2B). Cell-Matrix adhesions within the first 3 hours appear to be diffusively distributed on the cell membrane (Supplementary Fig. 2B inset). These observations support the simulation model of uniformly contracting circular cells.

Fiber alignment in the collagen bundles We have also quantified the fiber alignment of collagen bundles using coarse-grained nematic order parameter [4]. As shown in Supplementary Fig. 3, collagen bundles correspond to regions where collagen fibers are significantly more aligned than regions away from the bundles.

The mechanical origin of collagen bundles To demonstrate that collagen bundles can be created from purely mechanical means, we have developed a micro-stetcher using a programmable syringe pump (New Era). Two needle tips are submerged simultaneously in a collagen gel while the gel is formed. One metal needle is connected to the syringe pump which can be controlled by submicron range and the other needle tip is fixed. CellTak (Corning) is used to treat all contact

surfaces. Supplementary Fig. 5 and Supplementary Movie 2 show the device setup and confocal reflection recording of a typical experiment. In a typical experiment, we use microstretcher to deform a collagen matrix of concentration 1.5 mg/ml. Supplementary Fig. 5B1 and C1 show the unstressed configurations, where initial positions of two needle tips are indicated with red dashed circles. Collagen fibers are indicated in the green channel. Supplementary Fig. 5B2 and C2 show the configurations of the matrix deformed by moving one of the needles along the direction shown by the yellow arrows. After dwell time T_d as indicated in (B2) and (C2), the needles are moved back to their original positions. Short dwell time (10 min) allows the matrix to almost fully return to the initial configuration (Supplementary Fig. 5B3). Longer dwell time (1 hour) lead to irreversible reorganization of collagen fibers as highlighted in the white rectangle in Supplementary Fig. 5C3. Similar results can be found with higher collagen concentration (3.0 mg/ml) as shown in Supplementary Fig. 5D1-D3. In Supplementary Fig. 5D3 irreversible matrix remodelings are highlighted with white arrows.

We have also examined the effect of matrix metalloproteinase (MMP) on the formation of collagen bundles by applying a broad spectrum MMP inhibitor Marimastat ([5], Sigma-Aldrich) to the cells cultured in collagen matrix. Specifically, Marimastat is dissolved by 0.1% DMSO (volume ratio of DMSO and growth medium 1:100) into a final concentration of 0.5 μM. Right after cell-containing collagen gels solidify, we immerse the collagen gel with the Marimastat solution. After 10 hours, we imaged the sample and find presence of collagen bundles between most cell pairs within 80 μm distance. Supplementary Fig. 6 shows the confocal reflection images of representative collagen bundles.

Biophysical modeling of cell-induced matrix remodeling

Sliding events As we have mentioned in the main text, sliding events are not the actual sliding of the intersection points. Instead, they are dynamic breaks and reformations of the intersections points. The whole process is best illustrated by a sketch shown in Supplementary Fig. 7A.

To prevent intersection points from sliding indefinitely, we set a threshold $\Delta\gamma$ for the sliding event. Suppose there are three intersection points P_1, P_2, P_3 on fiber L_1, as shown in Supplementary Fig. 7B. The current length (rest length) of ith bond is represented by l_{ci} (l_{0i}), $i = 1, 2, 3, 4$. Initially, $l_{ci} = 1, l_{0i} = 1, i = 1, 2, 3, 4$ (Supplementary Fig. 7B, left). Suppose after the network is deformed, $l_{c1} = 1.1, l_{c2} = 1.2, l_{c3} = 0.4, l_{c4} = 1.2$ and sliding threshold is $\Delta\gamma = 0.2$ (Supplementary Fig. 7B, middle). The strain of each bond is $\gamma_i = (l_{ci} - l_{0i})/l_{0i}$. Thus $\gamma_1 = 0.1, \gamma_2 = 0.2, \gamma_3 = -0.6, \gamma_4 = 0.2$. Only when the strain difference between neighboring bond exceeds the sliding threshold $\Delta\gamma = 0.2$, will a intersection point slide. In this case, only P_2 and P_3 slide (Supplementary Fig. 7B, right). The sliding event set the new rest length of bonds proportional to their current length $l_{0i}^{new} = \frac{\sum_i l_{0i}}{\sum_i l_{ci}} l_{ci}$. Since P_1 does not move, $l_{01} = 1$ does not change. Sliding events change the rest length of other three bonds $l_{02} = \frac{1+1+1}{1.2+0.4+1.2} 1.2 = 9/7, l_{03} = \frac{1+1+1}{1.2+0.4+1.2} 0.4 = 3/7, l_{04} = \frac{1+1+1}{1.2+0.4+1.2} 1.2 = 9/7.$

Merging events For bonds merging events, we only consider those bond pairs that share a node at one end and decide whether to merge the bond paris into "super bonds". An example is shown

in Supplementary Fig. 7C. When the network is relaxed, all bonds (nodes) are far apart from each other (Supplementary Fig. 7C). As the network is deformed by contractile cells or external forces, some bonds may get close to each other (B_{ik} and B_{ij} in Supplementary Fig. 7C). Bond B_{ik} and B_{ij} merge into a "super bond" with probability $P_{merging}(d_{jk}, t) = \eta(d_0 - d) \cdot (1 - exp(-t/T_{merging}))$ within time span t (Supplementary Fig. 7C), where d is the distance between two neighboring bonds, d_0 the merging threshold and $T_{merging}$ the time scale of merging events.

Simulation scheme Our assumption here is that sliding events have a much faster time scale than merging events($T_{merging} \gg T_{sliding}$). We set $T_{sliding} = 0.5min, T_{merging} = 25min$ to reach good correspondence with experiments. Every half a minute the sliding events occur deterministically, while the merging events occur with probability $P_{merging}$. (We can also set sliding events to be probabilistic, but the results remain largely the same.) The dynamics of the system every $T_{sliding} = 0.5min$ can be approximately simulated with three steps:

Step 1: Minimize the free energy with conjugate gradient algorithm to get the equilibrium configuration of the network.

Step 2: Sliding events occurs. Update the rest length of all bonds involved in sliding events.

Step 3: Merging events occurs. Since merging events are on a much slower time scale, the occurrence of merging events is set to be probabilistic $P_{merging}(d, T_{sliding})$.

By repeating the three steps above we can get the configuration of the system every $T_{sliding} = 0.5min$.

Parameters The total energy (Eq (1)) is minimized by implementing the conjugate gradient algorithm. The parameters for the Triangular lattice-based model are bond occupation probability $p = 0.60$, network size $S = 60 \times 60$ for cell experiments and $S = 40 \times 40$ for bulk rheology, bending stiffness $\kappa = 10^{-3}$, stretching stiffness $k = 1$, sliding threshold $\Delta\gamma = 10^{-3}$, $T_{sliding} = 0.5min$, $T_{merging} = 25min$ and merging threshold $d_0 = 0.15$ unless stated otherwise.

We would like to comment on the choice of $T_{sliding}$ and $T_{merging}$. Our simulation makes a standard quasi-static assumption, i.e. the viscoelastic relaxation is much faster than the plastic relaxation, which has been confirmed by the experiments. In our model, $T_{sliding}$ is a sampling time. Every time step equals to $T_{sliding}$, we first minimize the elastic energy with a conjugate gradient algorithm, we then carry out the sliding events based on their computed probability. Therefore to make a comparison with experiments, we would like to keep $T_{sliding}$ much smaller compared with the plastic relaxation time, but at the same time lower bonded by the viscoelastic relaxation time. Our experimentally measured viscoelastic relaxation time is approximately 30 sec, 10 times smaller than the plastic relaxation time scale. We have therefore chosen Tsliding = 30 sec.

Our choice of $T_{merging}$ is also derived from the experiments. From **Fig 1** of the main text, we see the irreversibility of the system is significantly enhanced when the maturation time is increased from 0.2 hour to 1 hour, but less so after 1 hour. This observation suggests that $T_{merging}$ should be chosen between 0.2 hour and 1 hour. In fact, we find that varying $T_{merging}$ between 0.2 to 1 hour

only subtly changes the simulation results (Supplementary Fig. 12). We therefore set $T_{merging}$ = 25 min which fits experiments best.

We would also like to comment on the fact that the contraction factor β used in the simulation (40% to 50%) is much larger than typical experimentally meaured cell volume (area) change. Experimentally measured cell body compressive strain is typically 10%-30% [1], which is smaller compared with the β value we used. We think the discrepancy may come from two sources. One is dimensionality. The experimentally observed fiber densification has contributions from all three dimensions. The model, on the other hand, is 2D. To account for the same level of fiber densification, we expect a larger β has to be used. The other reason is that cells do not have to change volume in order to apply traction force. It is well known from 3D traction force microscopy that cells exert strong forces that are tangential to the cell membrane [2]. The tangential force can be generated by the relative shear between cells and the ECM. In our 2D model, however, traction force can only be generated by shrinking the area of the model cell. As a result, a larger β is necessary to reproduce the same level of fiber densification observed experimentally.

Additional simulation results of cell-induced collagen bundles As we have mentioned in the main text, contractile cells are modeled as circles. The initial radius of cells $R_0 = 6(bond\,length)$. The bond lengths of all bonds inside the circle are shortened isotropically by $\beta = 50\%$ (Radius of cells $a = R_0 \cdot \beta = 3(bond\,length)$. The distance between the center of cells $d = 22(bond\,length)$ in **Fig 2A**&**2B**. It's worth noting that sliding events alone is enough to induce fiber flux (or fiber densification) (Supplementary Fig. 8A), while the irreversibility is reduced to 20% at most without

merging event. Together with **Fig 2D** of the main text, we reach the conclusion that sliding events mainly contribute to fiber densification, while emerging event mainly contribute to irreversibility.

We also quantify the fiber alignment (Supplementary Fig. 8B) in collagen bundle in our simulation with nematic order parameter $\mathbf{Q} \equiv < \tilde{v}\tilde{v} - \frac{1}{d}I >$. Here $\tilde{v}$ is the unit vector pointing along the orientation of the bonds within the collagen bundle, and the average $< \cdots >$ is over all bonds within the collagen bundle. This measure was used in the experiments in the Ref [1]. We use a scalar, alignment order $q,\ 0 \leq q \leq 1$, to characterize the strength of the alignment. We define $q = [d/(d-1)\lambda_m$,where λ_m is the largest eigenvalue of $\mathbf{Q}$. We find the dynamic processes greatly enhance fiber alignment in collagen bundle in our simulation, which is verified by ttest (Supplementary Fig. 8B).

We verify our results in simulations with a different bond existence probability $p = 0.55$ instead of $p = 0.60$. Again the dynamic processes help fiber C. densification and D. alignment, showing our results are insensitive to the choice of the p value.

It's worth noting that we could have underestimated the influence of merging events. We only consider merging of bond pairs connected by a common node. In general, any two bonds that are close enough to each other can be merged into a ”super bond”. The number of merging events in our simulation gets significantly lower due to this reason. We can also increase the number of merging events by tuning parameters like merging threshold d_0 and merging time scale $T_{merging}$. The enhanced merging effects lower the degree of freedom in our model and make network stiffer, which considerably suppresses the flow of bonds, reduce $\Delta F/F$ in our simulation and strengthen

the irreversibility.

In the main text, we have only considered circular cells. This is supported by the experimentally measured distribution of cell aspect ratios (Supplementary Fig. 2). After the initial seeding period, however, some cells will elongate and can be better described by force dipoles. To account for these cells, we have considered elliptical cell shapes. As shown in Supplementary Fig. 9, elongated cells produce more pronounced collagen bundles, but the qualitative trends are consistent with circular cells.

Simulation of fiber network relaxation dynamics

Varying sliding and merging thresholds The dependence of macroscopic strain relaxation dynamics on the sliding and merging thresholds (which effectively controls the likelihood of these events) is shown in Supplementary Fig. 10. It turns out the merging events only affect the fitting coefficients slightly (Supplementary Fig. 10A, C &E). In stark contrast, sliding threshold $\Delta\gamma$ directly controls all the fitting coefficients (Supplementary Fig. 10B, D &F). In particular, when the sliding threshold $\Delta\gamma$ is large enough to prevent most sliding events, coefficient b drops to near zero, suggesting disappearance of strain relaxation. Such observation implies the strain relaxation is mostly attributed to sliding events.

Varying $T_{merging}$ time scale We have also investigated how the strain relaxation dynamics depend on the choice of $T_{merging}$. Supplementary Fig. 12 show that $T_{merging}$ is not a sensitive parameter.

Varying functional form of sliding probability In our model, the probability of sliding is a step function of the force controlled by the sliding threshold. On a coarse-grained sense, this is equivalent to the exponential form used in [3]. To demonstrate this, we have compared simulated relaxation dynamics using both models. As shown in Supplementary Fig. 11, the results are essentially the same.

Additional details of macroscopic strain relaxation measurements

rheology We monitor the gelation of each sample by measuring the shear modulus once per minute using a small amplitude oscillatory shear strain. We begin taking data immediately after the neutralized collagen solution is added to the pre-heated plates of the rheometer, and each sample gels for at least 90 minutes total. Supplementary Fig. 13A shows the shear modulus vs time during gelation for a typical collagen sample.

To characterize the nonlinear elasticity of a collagen gel, we measure the shear modulus versus strain amplitude for strains up to 100% (Supplementary Fig. 13B). All measurements were taken at a frequency of 1 Hz. Strain stiffening begins around 10% strain shown by an increase in the modulus. The modulus continuously increases with strain amplitude until around 60% strain. At this point the modulus suddenly decreases indicating the network yields. The vertical dashed line in Supplementary Fig. 13B shows 20% strain, the magnitude used for the experiments on recovery versus dwell time described in the main text. This strain is just inside the beginning of the nonlinear elastic region.

Fitting for relaxation time constants Of the two time constants characterizing the strain recovery of collagen gel, only the larger time constant, τ_p should depend on the dwell time. The smaller time constant, τ_v, should be the same for all recovery curves. To find a single τ_v which best fits the data, we fix τ_v to be the same for all dwell times, fit each recovery curve for the remaining parameters, and then calculate the total mean squared error. We define the total mean squared error (MSE_{tot}) as the sum of the individual mean squared error for each dwell time. So $MSE_{tot} = \sum MSE_{T_d}$ and $MSE_{T_d} = \frac{1}{n}\sum_{i=1}^{n}(\hat{\varepsilon}_i - \varepsilon_i)^2$, where T_d is the dwell time, ε is the data and $\hat{\varepsilon}$ is the fit. By repeating

this for many different τ_v, we find a single global τ_v which best fits all the data. Supplementary Fig. 14 shows the total mean squared error versus τ_v for a typical experiment. The red circle in the figure shows the chosen τ_v, which does indeed give a global minimum for the error.

History-dependent relaxation tested with independent samples We also ran experiments in which a new sample was used for each dwell time so that no residual strain or alignment of fibers from previous strains will affect the relaxation dynamics. Supplementary Fig. 15A shows the curves for relaxation from a 20% strain applied for five different dwell times from 1 to 20 minutes. No global τ_v was used due to every experiment being run on a new sample. Instead, each recovery curve was independently fit for both time constants. The experiment was run on three independent samples for each dwell time. Supplementary Fig. 15B shows the second time constant τ_p, and Supplementary Fig. 15C shows the residual strain ε_r averaged over the three experiments. Error bars show the standard deviation. The results are qualitatively the same as when a single sample was used for each dwell time, with the second time constant and the residual strain both increasing with dwell time.

History-dependent relaxation tested with independent samples and extended relaxation time While 15 minutes of relaxation is sufficient to show the dependence of the residual strain with respect to the dwell time, we have further extended to longer relaxation time to examine if the trends still holds. Indeed, we find that residual strains obtained by fiting 25 minutes of relaxation also increase with dwell time (Supplementary Fig. 16A). This is not surprising, because τ_p is about 5 minutes, therefore 15 minutes is sufficient to cover the most dynamic part of strain relaxation.

Supplementary Fig. 16B further confirms that strain relaxation is very slow past 15 minutes.

Plasticity of fibrin gels We have also tested the history-dependet relaxation of fibrin gels, and find qualitative agreement with collagen gels. To prepare the fibrin gels, we mix 2 mg/mL human fibrinogen (in 50 mM TrisHCl buffer, containing 150 mM NaCl and 10 mM CaCl2), with human thrombin to a final concentration of 0.6 U/mL. As shown in Supplementary Fig. 17A, fibrin show nonlinear elasticity around 30% shear strain. Similar to the experiments reported for collagen matrices, we measure the strain relaxation dynamics (Supplementary Fig. 17B) of a fibrin gel with initial strain of 50% hold for various dwell times. To identify the plastic relaxation time τ_p, we fit the later portion ($t \geq 70$ sec) of the strain relaxation with single exponential and obtain τ_p as the negative inverse of the exponent. In the case of fibrin gels, excessive underdamped oscillations in the early stage of the relaxation ($t \leq 70$ sec) make it impractical to use double exponential fitting, as we do for collagen gels, for the entire curves. We find that both τ_p and residual strain increase at longer dwell time, as shown in Supplementary Fig. 17C-D. Therefore fibrin gels, like collagen matrices, demonstrate mechanical plasticity.

Additional details of micromechanical compliance

We have measured the micromechanical compliance of collagen matrices with holographic optical tweezers. Supplementary Fig. 18 illustrates the definition of a directional compliance based on the particle displacement caused by the optical trap.

Supplementary Figures

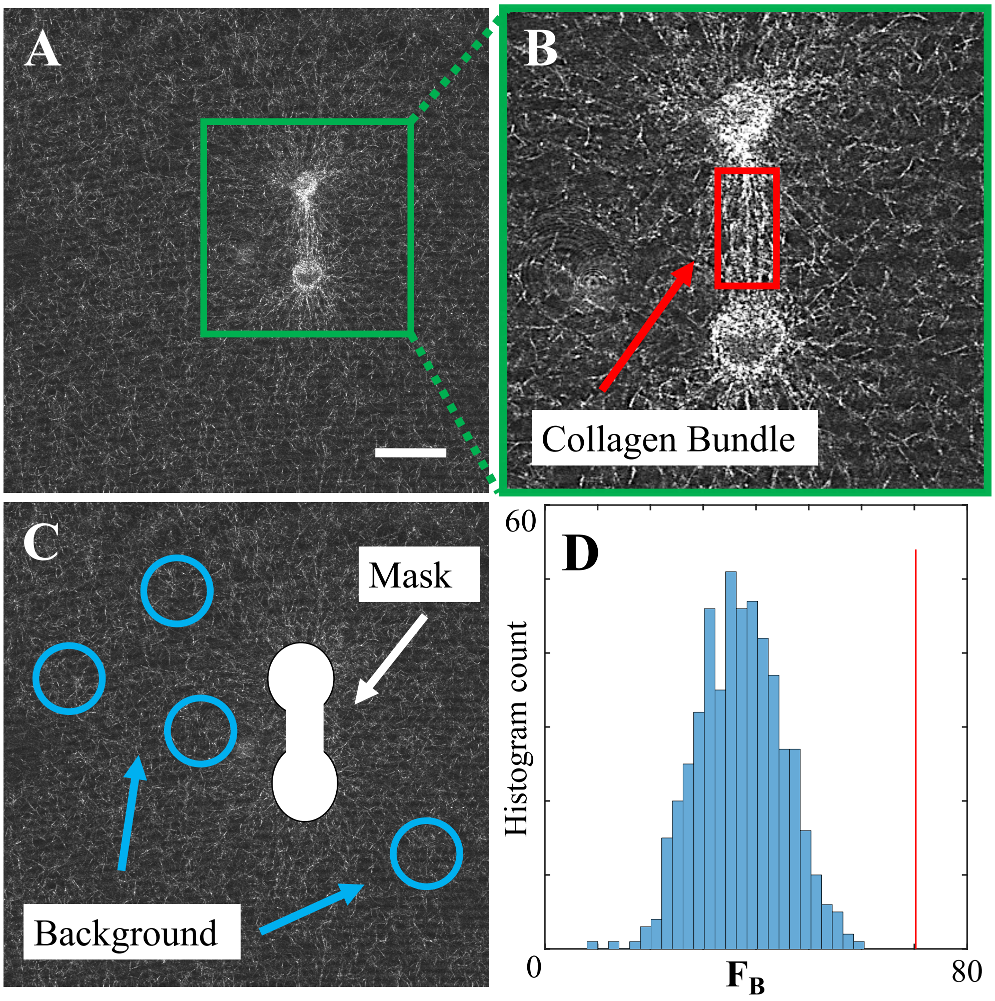

Supplementary Figure 1: Quantitative analysis of collagen bundles from confocal reflection images. (**A**) A sample confocal reflection image Scale bar is 50 μm. (**B& C**) Image analysis to quantify the mean intensity of the collagen bundle F_b and the background intensity distribution $\{F_i\}$. See Supplementary Methods for details. (**D**) The histogram of F_i shows an approximate Gaussian distribution with mean value 37, and standard deviation of 12. Intensity of the collagen bundle $F_b = 70$ (red line) is evidently distinguishable from the background.

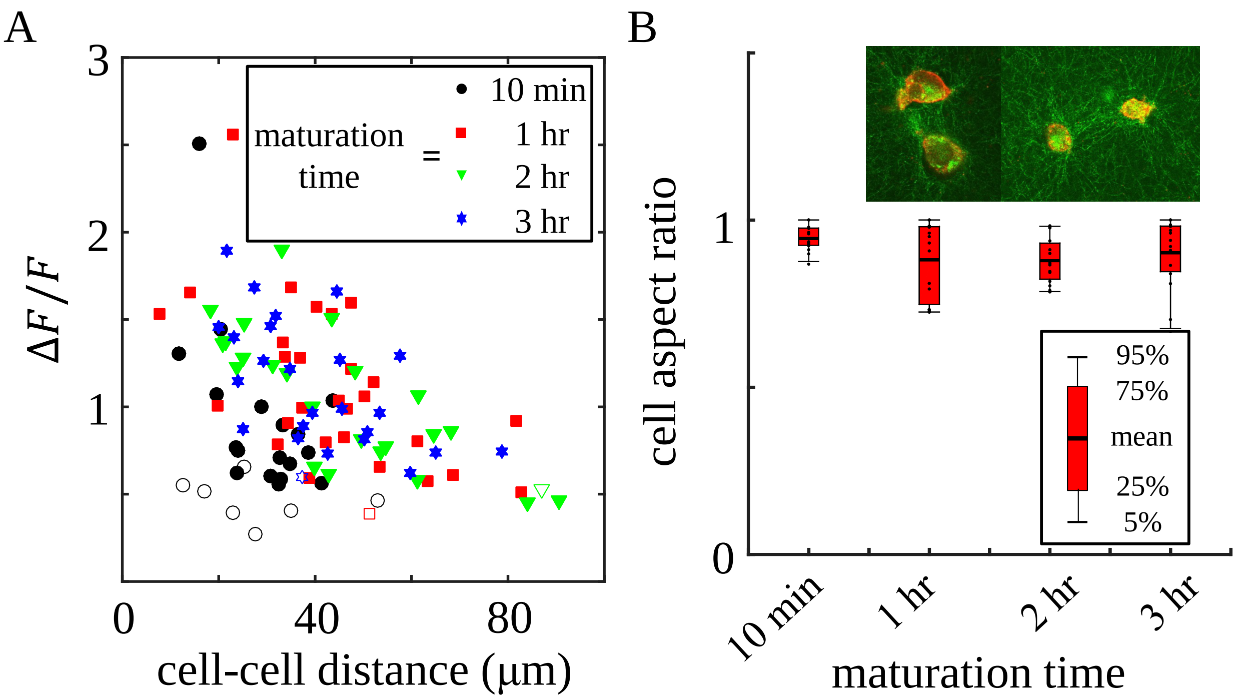

Supplementary Figure 2: Additional experimental results of collagen bundles formed by cell pairs. (**A**) $\Delta F/F$ decreases when the cell-cell distance increases. The data points are distinguished by their signal to noise ratios. Closed symbols represent $\Delta F/F > 3\delta F/F$ (corresponding to $p < 0.0027$), and open symbols represent $\Delta F/F < 3\delta F/F$. Here δF is the background fluctuation that follows closely to Gaussian distribution. (**B**) Cell morphology characterized by the aspect ratio of their 2D projection at various maturation times. Inset: confocal images of cells with fluorescently labeled vinculin (red) in the collagen matrices (green).

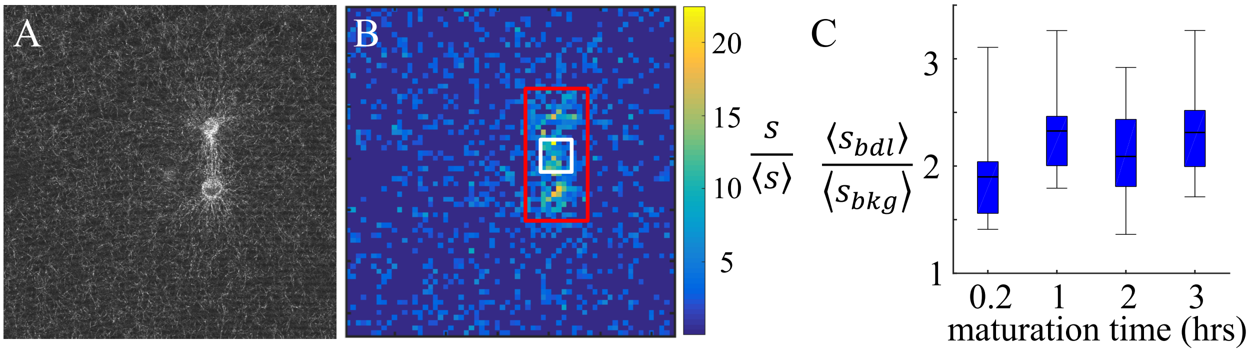

Supplementary Figure 3: Collagen bundles consist of highly aligned fibers. We have quantified the fiber alignment using coarse-grained nematic orders as in [4]. Briefly, an confocal reflection image as shown in (**A**) is first converted to a binary image using Otsu threshold, then divided into subwindows of 32 X 32 pixel^2 or approximately 10 X 10 μm^2. Within each subwindow, we have calculated the average nematic order $s = |\langle e^{2i\theta} \rangle|$. s is upper bonded to 1, in the case all lines (fibers) are in the same direction; or lower bonded to 0, in the case lines are randomly aligned. (**B**) shows the coarse-grained nematic field s corresponding to (**A**), where s is normalized by the average of the full field of view (subwindows that are purely background considered as zero). To further compare the level of alignment in the bundle region and away from the bundles, we have calculated the relative nematic order $\frac{\langle s_{bdl} \rangle}{\langle s_{bkg} \rangle}$. Here $\langle s_{bdl} \rangle$ is the average over the bundle region (within the white box of (**B**)). $\langle s_{bkg} \rangle$ is the average over the background region (outside of the red box of (**B**)). Subwindows of purely background are excluded from $\langle s_{bdl} \rangle$ and $\langle s_{bkg} \rangle$. We have calculated all experimental images corresponding to **Fig. 1D** of the main text, and the result is shown in (**C**).

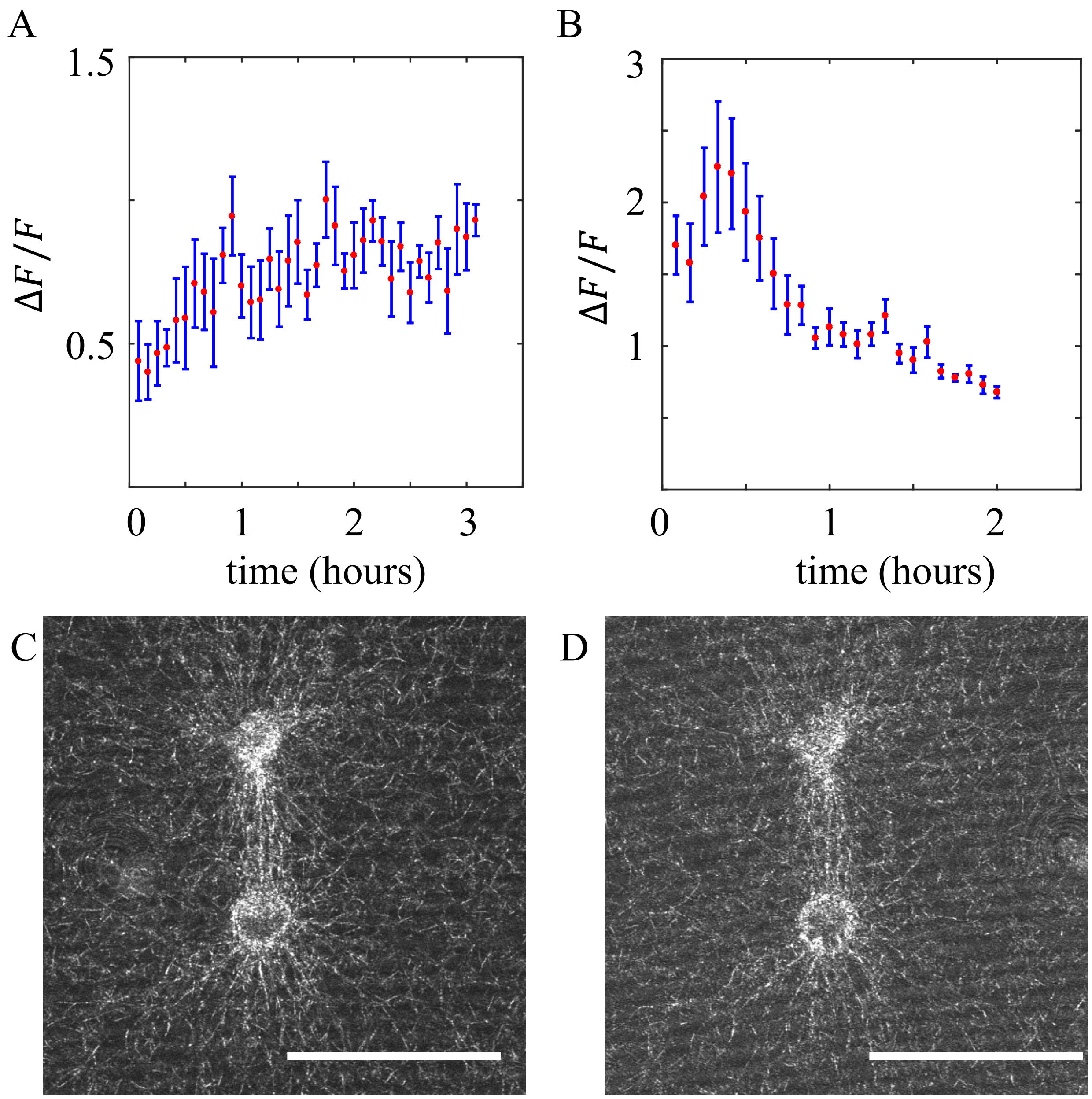

Supplementary Figure 4: Examples showing the temporal evolution of relative intensity of collagen bundles. (**A**) $\Delta F/F$ during maturation time of a collagen bundle. (**B**) $\Delta F/F$ of a collagen bundle during Cytochalsin-D treatment. (**C**) A confocal reflection image showing a collagen bundle before treating with cytochalsin-D. Scale bar is 50 μm. (**D**) A confocal reflection image of the same region as in (A) after treating with cytochalsin-D. It is clear that the collagen bundle persists even after relaxing the cellular traction force.

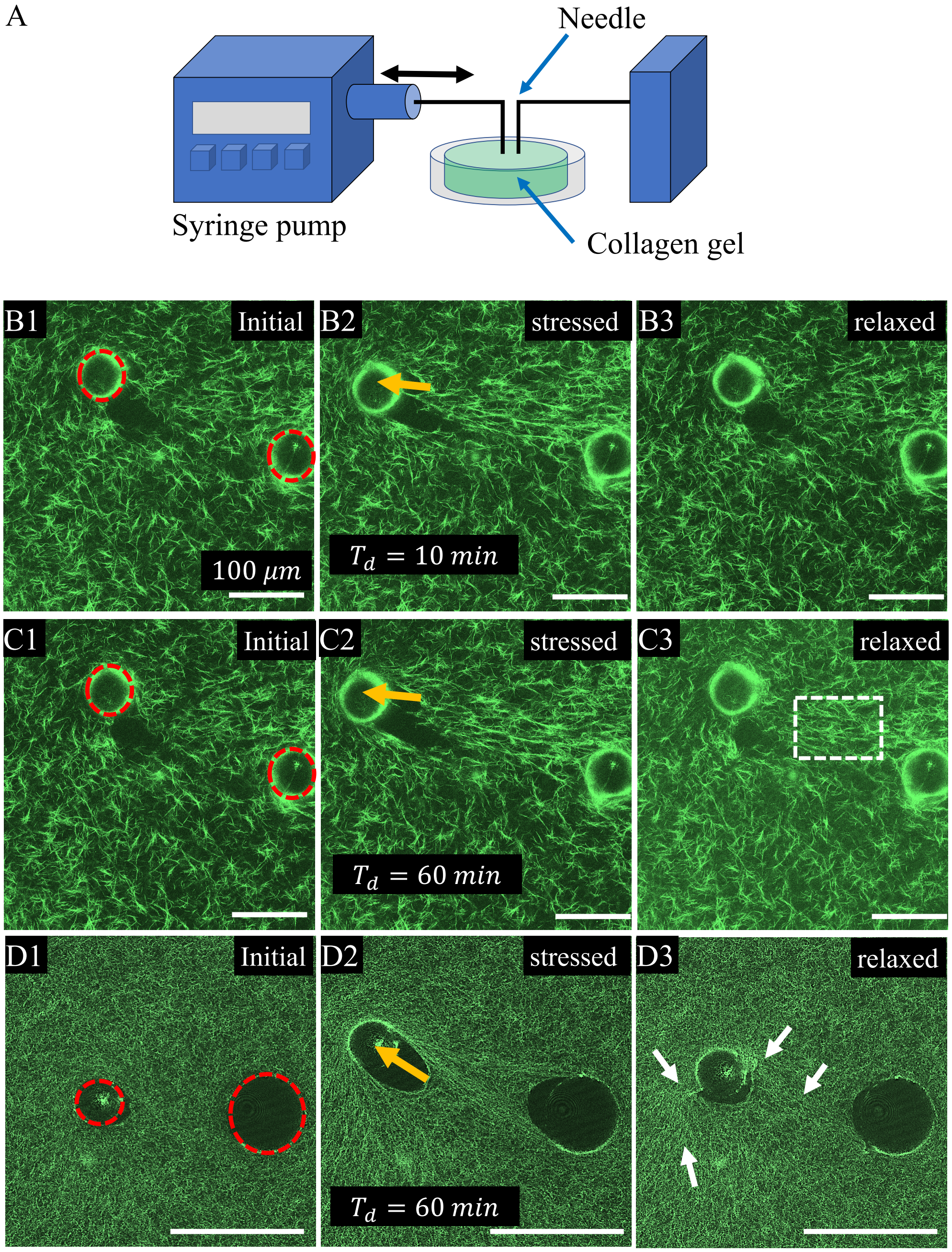

Supplementary Figure 5: Micromechanical stretch irreversibly induces formation of collagen bundles. **(A)** Schematic diagram of the experimental setup. **(B& C)** Confocal reflection images showing collagen matrix (1.5 mg/ml) remodel depends on dwell time. **(D)** Confocal reflection images showing irreversible collagen matrix (3 mg/ml) remodel with dwell time of 1 hour. See Supplementary Methods for more details.

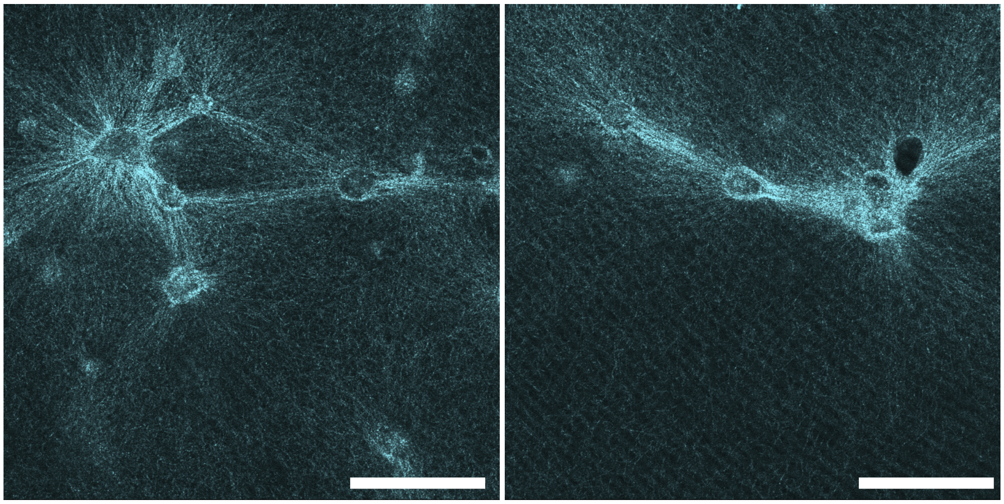

Supplementary Figure 6: Representative confocal reflection images showing the collagen bundles between cells treated with Marimastat, a broad spectrum MMP inhibitor. Scale bars: 100 μm.

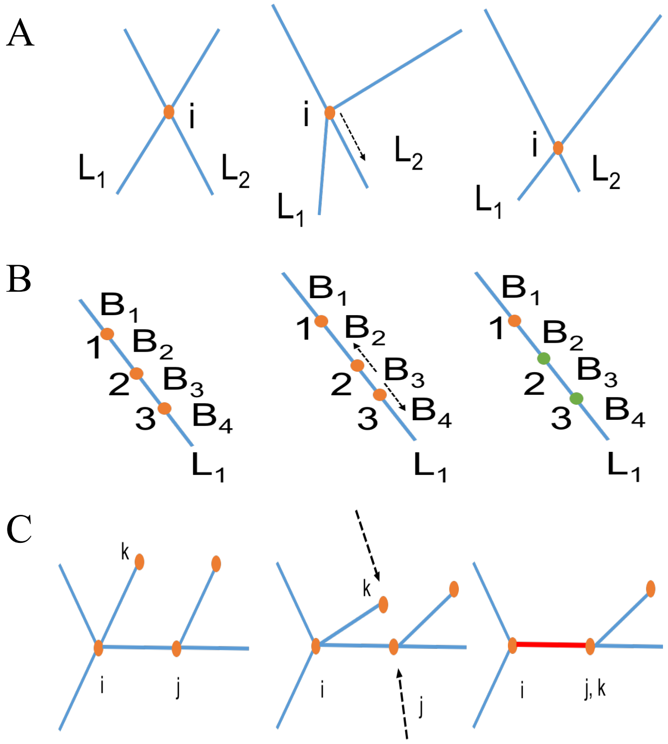

Supplementary Figure 7: Schematics showing key steps in the simulation. **(A)** A sketch of intersection point "sliding". Two fibers L_1,L_2 intersecting at point i. For simplicity, only two bonds of each fiber are shown. Left: two fibers in the relaxation state. Mid: Fibers L_1,L_2 are stretched and bended by surrounding fibers (not shown). Note fiber L_2 is not necessarily straight as shown in the figure. Intersection point i receives a finite pulling force (dash line) from fiber L_1. Right: The intersection point i first break and then reform at a different location. **(B)** An example for intersection point "sliding" along fiber L_1. **(C)** An example for two bonds merging into a "super bond".

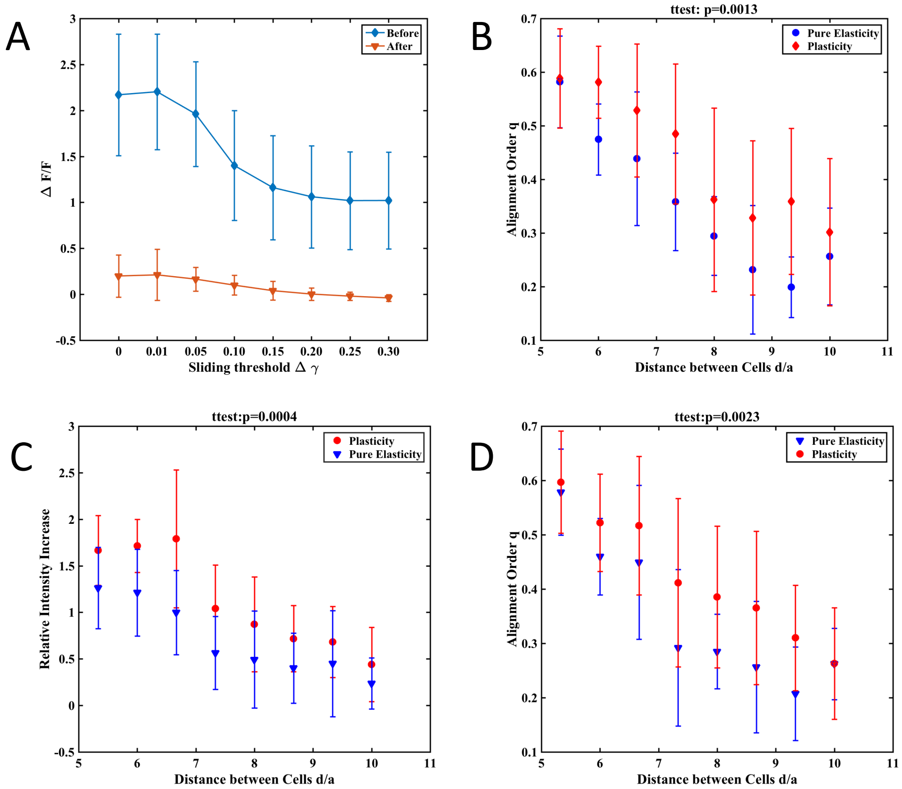

Supplementary Figure 8: Additional simulation results of collagen fiber densification between two contracting circular cells. **(A)** Simulation without merging events. Before (blue) and after (orange) releasing cell contractility.**(B)** The dynamic processes of sliding and merging help fiber alignment in collagen bundle. All parameters are the same to **Fig. 2C** in the main text. **(C& D)** Simulation results with a lower p=0.55 instead of 0.60. Again the dynamic processes help fiber C. densification and D. alignment, showing our results are insensitive to the choice of p value.

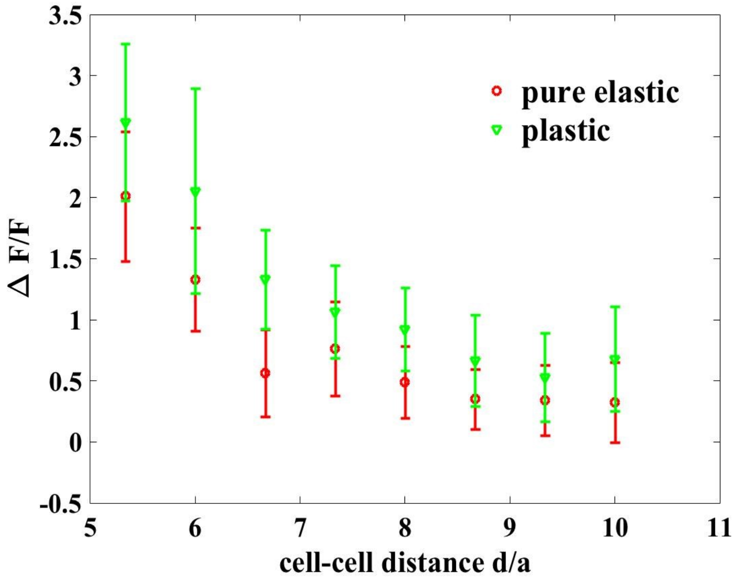

Supplementary Figure 9: Simulation results of collagen fiber densification between two contracting elliptical cells which mimic force dipoles. Simulation parameters are the same as those in Fig. 2C of main text, except that the cells are ellipsoids with major axis of 6 and minor axis of 3.

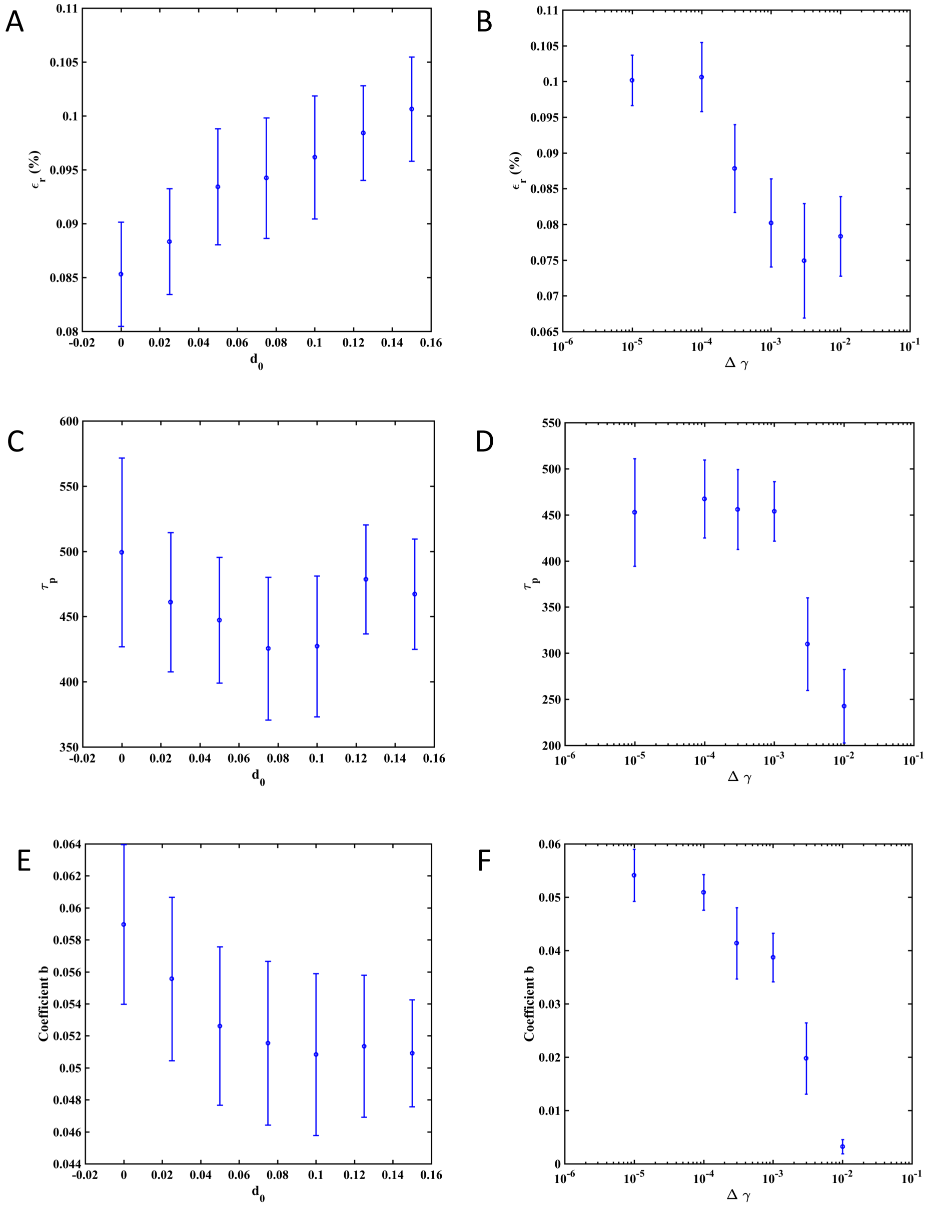

Supplementary Figure 10: The simulated strain relaxation dynamics with varying merging and sliding thresholds. Simulation results are fitted with function $\epsilon(t) = be^{-t/\tau_p} + \epsilon_r$. **(A,C& E)** show the residual strain, time constant and coefficient b at varying merging threshold d_0. **(B,D& F)** show the residual strain, time constant and coefficient b at varying sliding threshold $\Delta\gamma$.

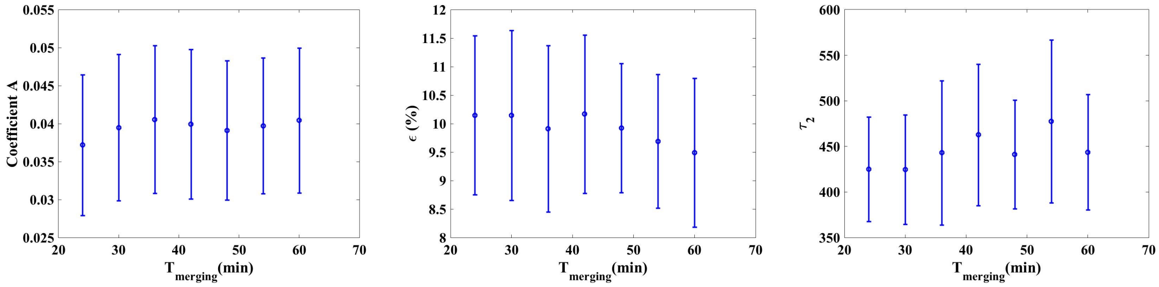

Supplementary Figure 11: The simulated strain relaxation dynamics with different $T_{merging}$. The results show that $T_{merging}$ is not a sensitive parameter for the model.

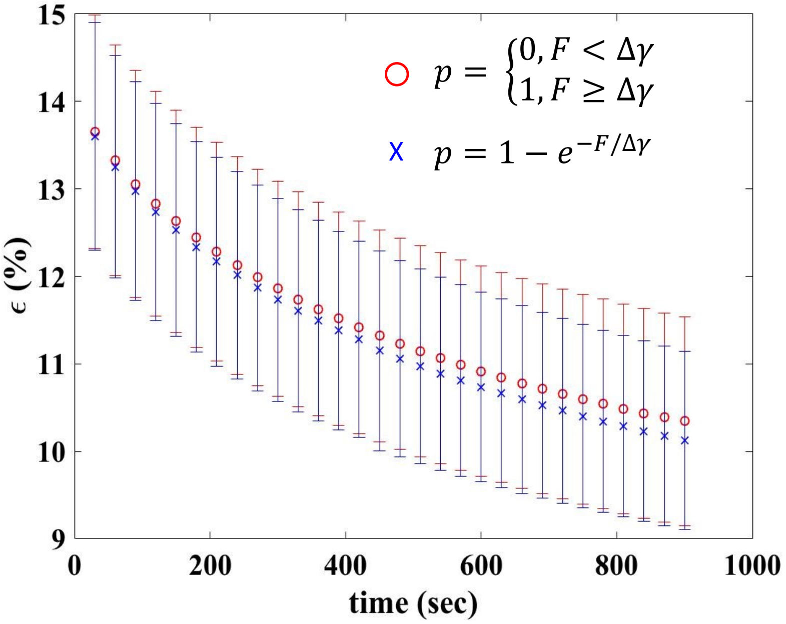

Supplementary Figure 12: The simulated strain relaxation dynamics with different forms of sliding probability p as a function of force F.

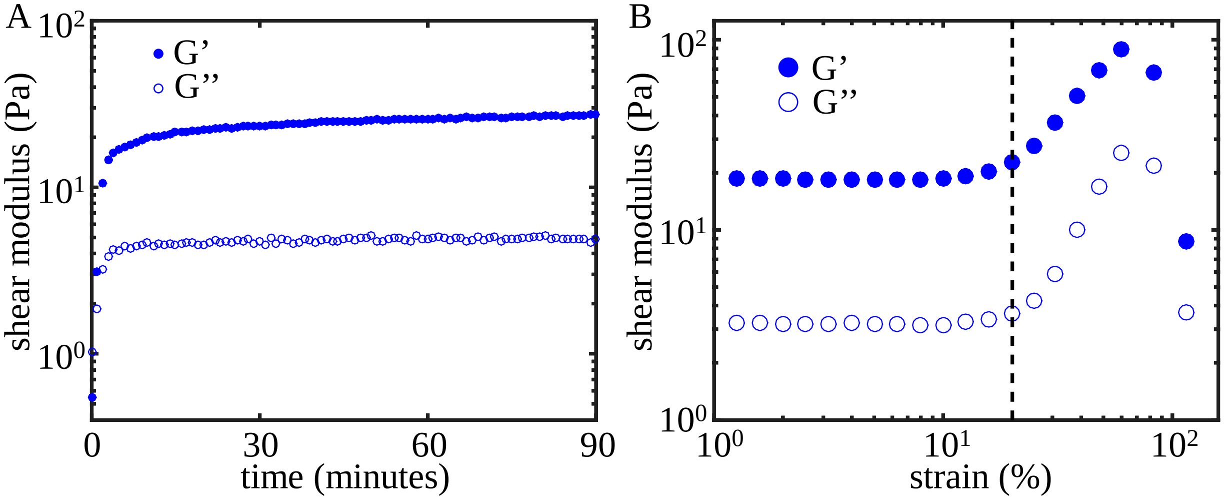

Supplementary Figure 13: Collagen rheology. **(A)** Temporal characterization of the gelation process for a typical collagen gel. The complex shear modulus is measured once per minute using a 0.5% oscillatory strain with a frequency of 1 Hz and the total gelation time is 90 minutes. **(B)** Shear modulus versus strain amplitude. Strain stiffening begins to appear around 10% strain and the sample yield is around 60%. The vertical dashed line shows 20% strain.

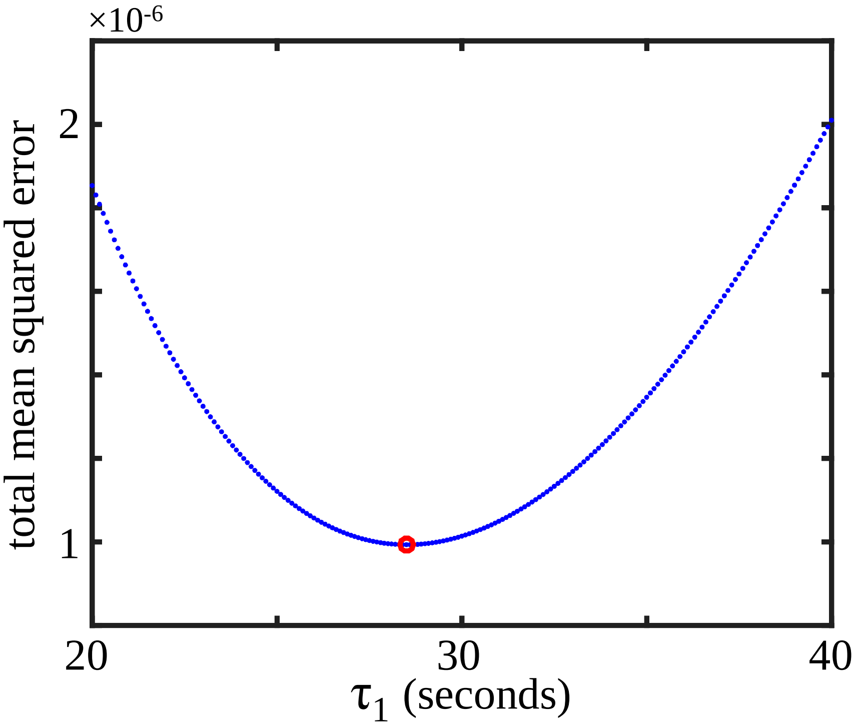

Supplementary Figure 14: Global fitting for τ_v. Total mean squared error (MSE_{tot}) verses global τ_v. The red circles shows the final τ_v gives a minimum in MSE_{tot}.

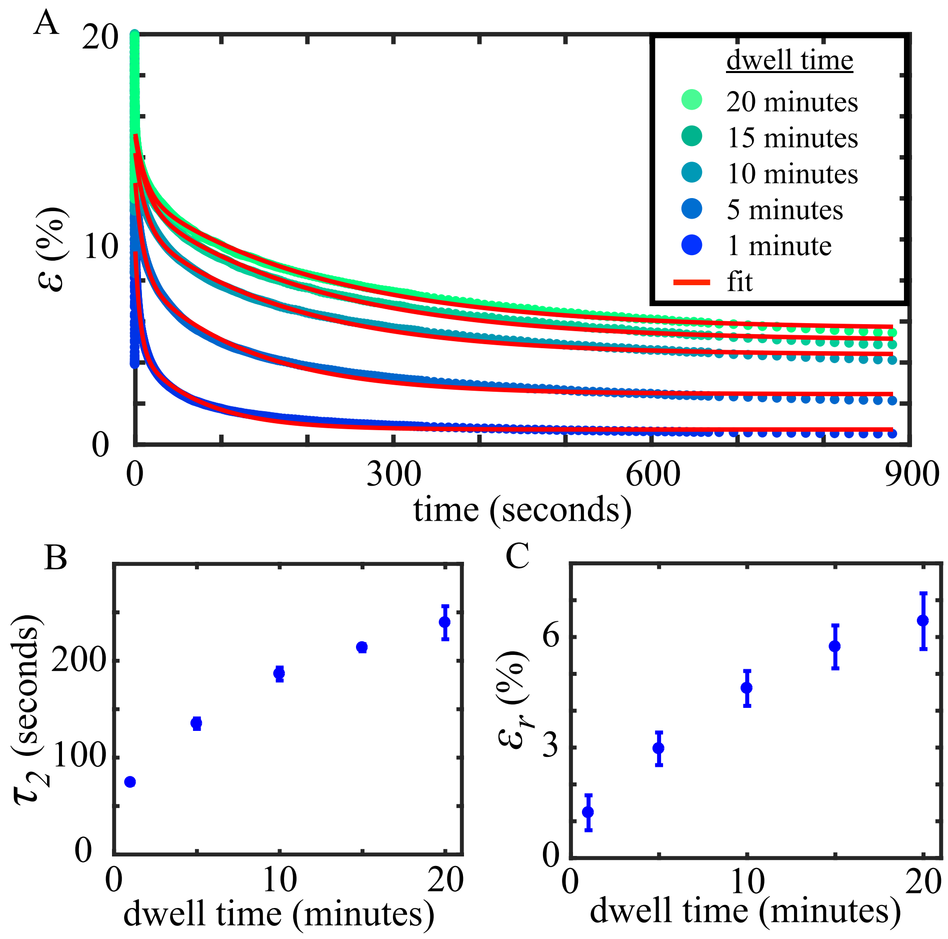

Supplementary Figure 15: Characterization of relaxation dynamics when a new sample is used for every dwell time. **(A)** Strain recovery curves from 20% strain applied for 1, 5, 10, 15, and 20 minutes. All curves are from different samples which had not been previously strained. **(B)** Second time constant τ_2 versus dwell time. **(C)** Residual strain ε_r versus dwell time. Error bars show standard deviation of three independent experiments.

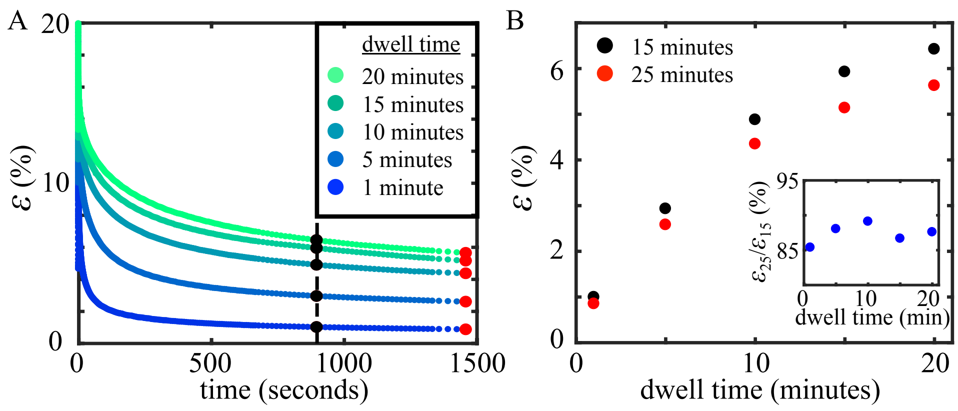

Supplementary Figure 16: Strain relaxation dynamics recorded over 25 minutes. **(A)** Strain relaxation from 20% initial strain applied for 1, 5, 10, 15, and 20 minutes of dwell time. All curves are from different samples. Black circles show strain at 15 minutes and red circles show strain at 25 minutes. (B) Strain at 15 and 25 minutes versus dwell time. Inset: Ratio of the strain at 25 minutes over the strain at 15 minutes.

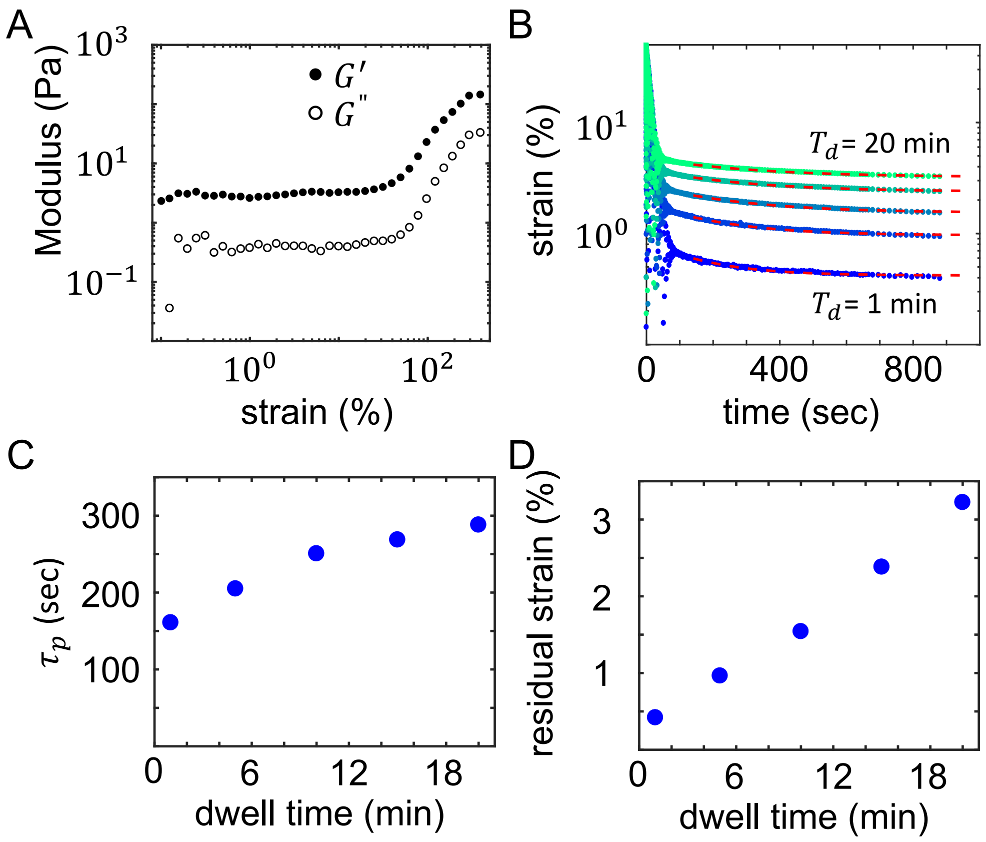

Supplementary Figure 17: The history-dependent strain relaxation of fibrin gels. **(A)** The storage and viscous moduli of fibrin gel at various strains measured at 1 Hz. **(B)** The strain relaxation dynamics following dwell time of 1, 5, 10, 15, and 20 minutes with initial shear strain of 50%. Dashed lines: single exponential fits of the curves from $t \geq 70$ sec. **(C)** The plastic relaxation time obtained from the fittings of (B) at varying dwell time. **(C)** The residual strains at varying dwell time.

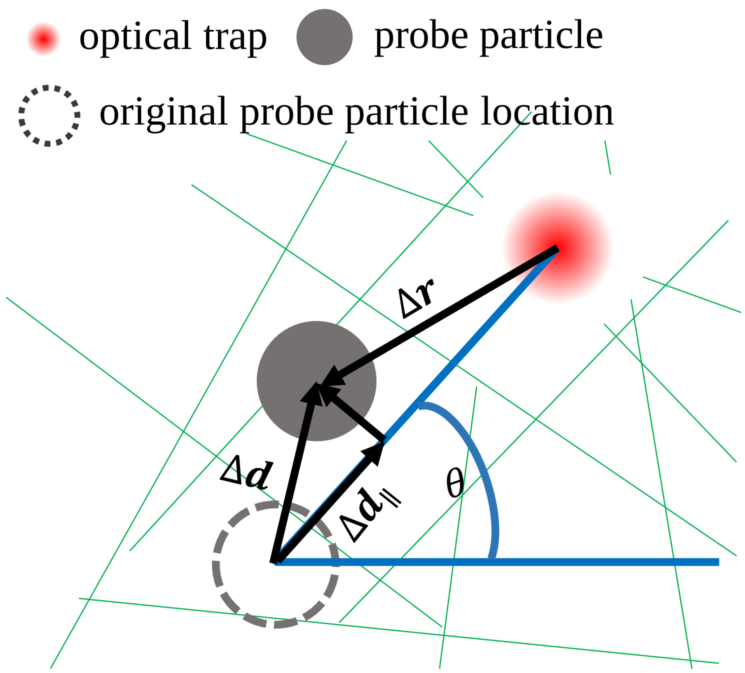

Supplementary Figure 18: To measure the directional compliance $J(\theta)$, we perturb a probe particle from its original position $\mathbf{r}_0$ (dashed circle) with an optical trap centered in the same focal plane of the particle but displaced from $\mathbf{r}_0$ in the direction of θ by $\Delta r = 0.7\mu$m. The particle equilibrates at a new position (gray disk) as a result of balance between optical force and matrix elastic restoring force. Notice that due to the nonlinear elasticity of collagen matrices, particle displacement $\Delta\mathbf{d}$ is not along the direction of θ. The projection of $\Delta\mathbf{d}$ in the trap direction is $\Delta\mathbf{d}_{\|}$. With the trap stiffness measured to be $k = 0.5\ pN/\mu$m, and particle radius $a = 1.5\mu$m, the directional compliance is defined as $J(\theta) = 6\pi a \frac{|\Delta\mathbf{d}_{\|}|}{k(|\Delta\mathbf{r}|-|\Delta\mathbf{d}_{\|}|)}$. For linear elastic materials, this definition is equivalent to the standard definition of elastic compliance.

Supplementary References

[1] M.S.Hall *et al.* Fibrous nonlinear elasticity enables positive mechanical feedback between cells and ecms. *Proc. Natl. Acad. Sci.* **113**, 14043 (2016).

[2] WR, L. *et al.* Measurement of mechanical tractions exerted by cells in three-dimensional matrices. *Nat. Methods* **113**, 969 (2010).

[3] Nam, S., Hu, K. H., Butte, M. J. & Chaudhuri, O. Strain-enhanced stress relaxation impacts nonlinear elasticity in collagen gels. *Proc. Natl. Acad. Sci.* **113**, 5492 (2016).

[4] Jones, C., Liang, L., Lin, D., Jiao, Y. & Sun, B. The spatial-temporal characteristics of type I collagen-based extracellular matrix. *Soft Matter* **10**, 8855 (2014).

[5] Brafman, D. A., Phung, C., Kumar, N. & Willert, K. Regulation of endodermal differntiation of human embryonic stem cells throuh integrin-ecm interactions. *Cell Death Differ.* **20**, 369 (2013).
