# Supplementary material for: Stress-induced plasticity of dynamic collagen networks
Source: Nat Commun. 2017 Oct 10;8:842. doi: 10.1038/s41467-017-01011-7 (PMC5635002; doi:10.1038/s41467-017-01011-7)
Supplement: Supplementary file 3 — Description of Additional Supplementary Files [file 41467_2017_1011_MOESM3_ESM.pdf]

Description of Additional Supplementary Files

File Name: Supplementary Movie 1
Description: Bright field imaging of a pair of MDA-MB-231 cells after seeding in a 3D collagen matrix

File Name: Supplementary Movie 2
Description: Reflection imaging of a pair of MDA-MB-231 cells after seeding in a 3D collagen matrix.

File Name: Supplementary Movie 3
Description: Reflection imaging of a microstretcher experiment with dwell time of 10 minutes.

File Name: Supplementary Movie 4
Description: Reflection imaging of a microstretcher experiment with dwell time of 1 hour.
